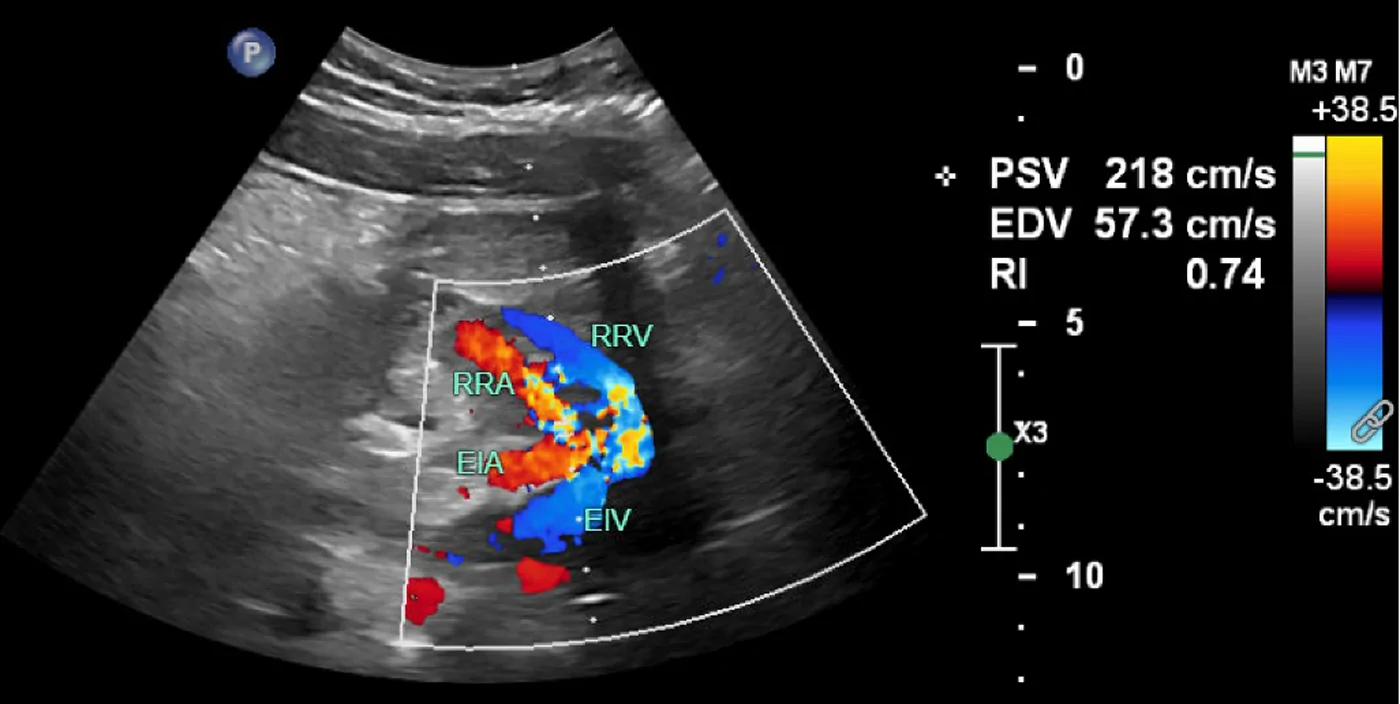# Autotransplantation for circum-aortic left renal vein compression after failed left renal vein transposition

**DOI:** 10.1016/j.jvscit.2026.102506

**Published:** 2026-09-07

**Authors:** Julia Rosenberg, Jane Quackenbush, Georges Jreij, Langford Green, Eleanor Dunlap, Justin Robbins, Khanjan Nagarsheth

**Affiliations:** a Division of Vascular Surgery, Department of Surgery, University of Maryland School of Medicine, Baltimore, MD; b Division of Vascular and Endovascular Surgery, Department of Surgery, Saint Louis University School of Medicine, St. Louis, MO

**Keywords:** Nutcracker syndrome, Left renal vein compression, Vascular surgical procedures, Renal autotransplantation

## Abstract

Circum-aortic nutcracker syndrome is a rare form of left renal vein compression with limited evidence to guide management. We present two sibling patients with complex renal vein anatomy who had persistent symptoms despite initial left renal vein transposition. Both underwent renal autotransplantation after recurrence of flank and abdominal pain, with imaging demonstrating ongoing compression and collateralization. Following autotransplantation, both patients experienced sustained symptom resolution and preserved renal function at follow-up.

Nutcracker syndrome (NCS) is characterized by compression of the left renal vein (LRV) resulting in renal venous hypertension and development of collateral circulation due to impaired blood flow. The most common anatomical variant involves LRV entrapment between the aorta and the superior mesenteric artery (SMA), known as anteaortic NCS. Less commonly, posterior NCS occurs when a retroaortic or circumaortic LRV is compressed between the aorta and the spine.^1^, ^2^, ^3^ Symptoms are often complex and include left flank or pelvic pain, hematuria, and proteinuria. The severity of symptoms typically guides management, where LRV transposition is often considered the preferred treatment; however, its efficacy in directly addressing compression in the setting of LRV variants is not well understood.^4^, ^5^, ^6^ Here we describe two siblings with NCS in the setting of circumaortic LRV with consent obtained from both patients and Institutional Review Board approval. Both patients underwent renal autotransplantation as a secondary intervention following persistent symptoms after initial LRV transposition.

## Case 1

A 20-year-old woman with a history of postural orthostatic tachycardia syndrome, hypermobile Ehlers-Danlos syndrome (hEDS), and neurogenic thoracic outlet syndrome presented with chronic abdominal pain and was evaluated for possible venous compression. Mesenteric arterial duplex ultrasound showed a narrow SMA angle and elevated celiac velocities consistent with median arcuate ligament syndrome (MALS). Computed tomography (CT) determined a SMA angle of 12.1 degrees. She underwent a venogram with intravascular ultrasound (IVUS), which revealed circumaortic anatomy of the LRV with compression of the anteaortic LRV (Fig 1). Based on these findings, the patient underwent open MALS release and ligation of the retroaortic LRV followed by transposition of the anteaortic LRV. Significant collateralization from the LRV to the ovarian and the adrenal veins was noted.

**Fig 1:**
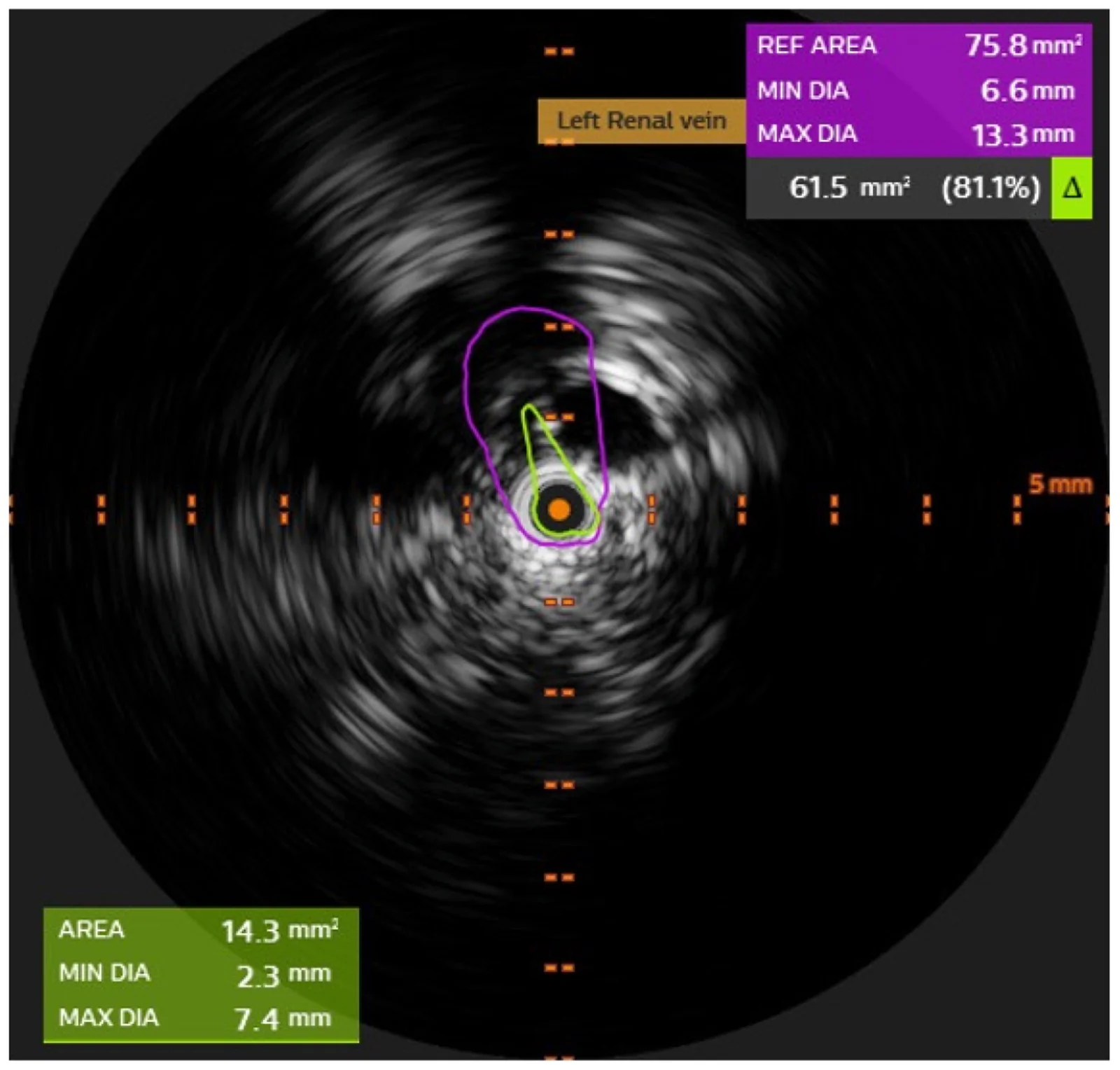
Preoperative intravascular ultrasound (IVUS) image in patient 1 demonstrates compression of the anterior left renal vein.

Six months later, the patient presented with recurrent symptoms consistent with NCS. A repeat venogram (Fig 2, *A*) with IVUS (Fig 2, *B*) showed narrowing of the anteaortic LRV, multiple collateral veins from the renal hilum to retroperitoneal veins, and internal iliac reflux. Given these findings and symptoms concerning for recurrent compression, we offered left renal autotransplant as definitive treatment.

**Fig 2:**
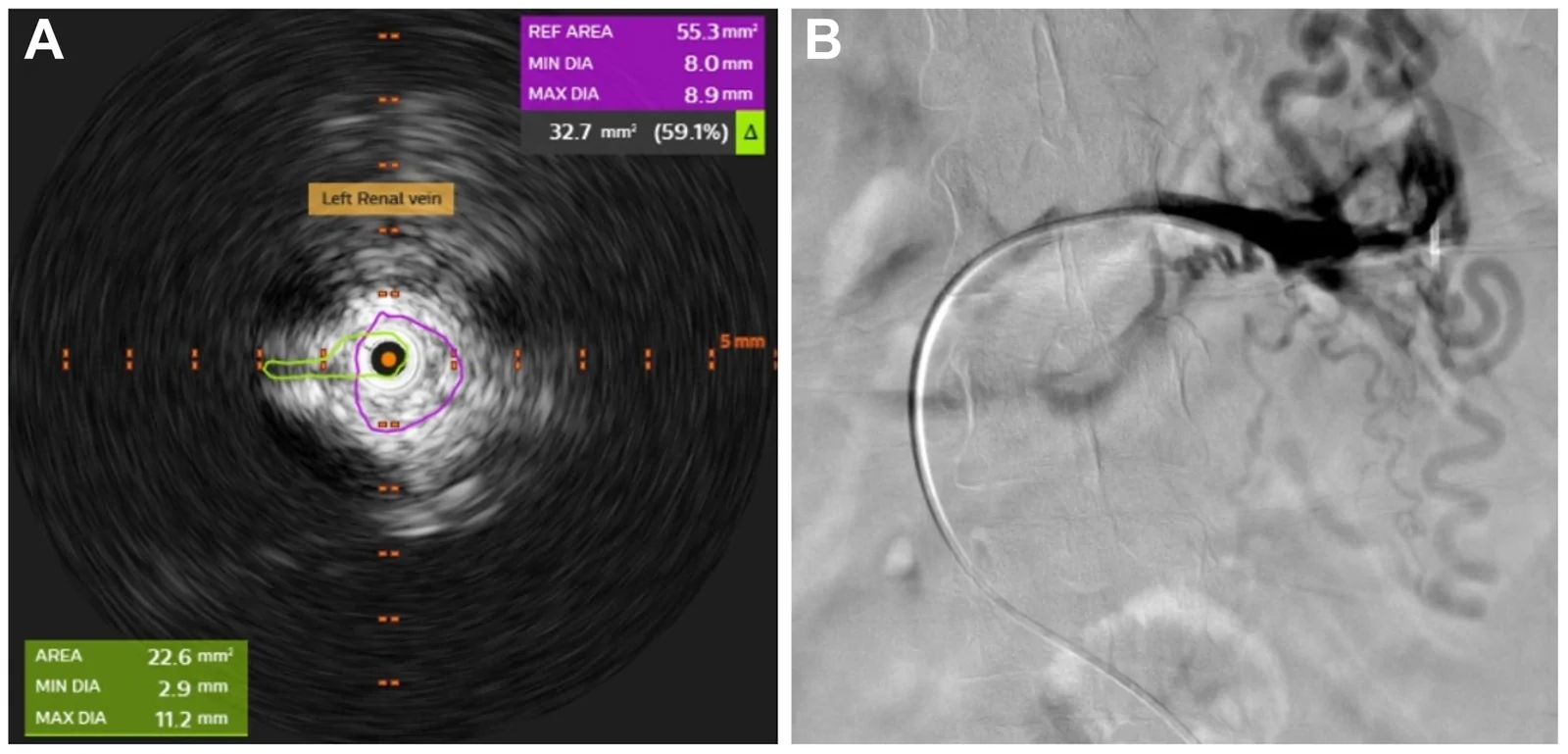
Post-left renal vein (LRV) transposition imaging findings in patient 1. **A,** Intravascular ultrasound (IVUS) demonstrates stenosis of the LRV. **B,** Venography reveals extensive collateralization from the renal hilum to the ovarian vein and circumaortic LRV.

In our institution, this is performed collaboratively by vascular, transplant, and urologic surgery services. Following laparoscopic left nephrectomy, the resected kidney is then prepared on the back table with removal of Gerota’s fascia and skeletonization of the renal vein, artery, and ureter. The kidney is then autotransplanted into the right iliac fossa. Venous and arterial anastomoses were completed by vascular service, and ureteral reimplantation was performed with transplant service. There was immediate kidney reperfusion and adequate urine production (Fig 3, *A* and *B*). The patient recovered well postoperatively, and duplex imaging demonstrated patent renal vasculature. At 15 months postsurgery, renal function remained preserved with stable serum creatinine of 0.69 mg/dL compared with 0.68 mg/dL preoperatively, and the patient reported complete resolution of compression-related abdominal pain (Fig 3, *C*).

**Fig 3:**
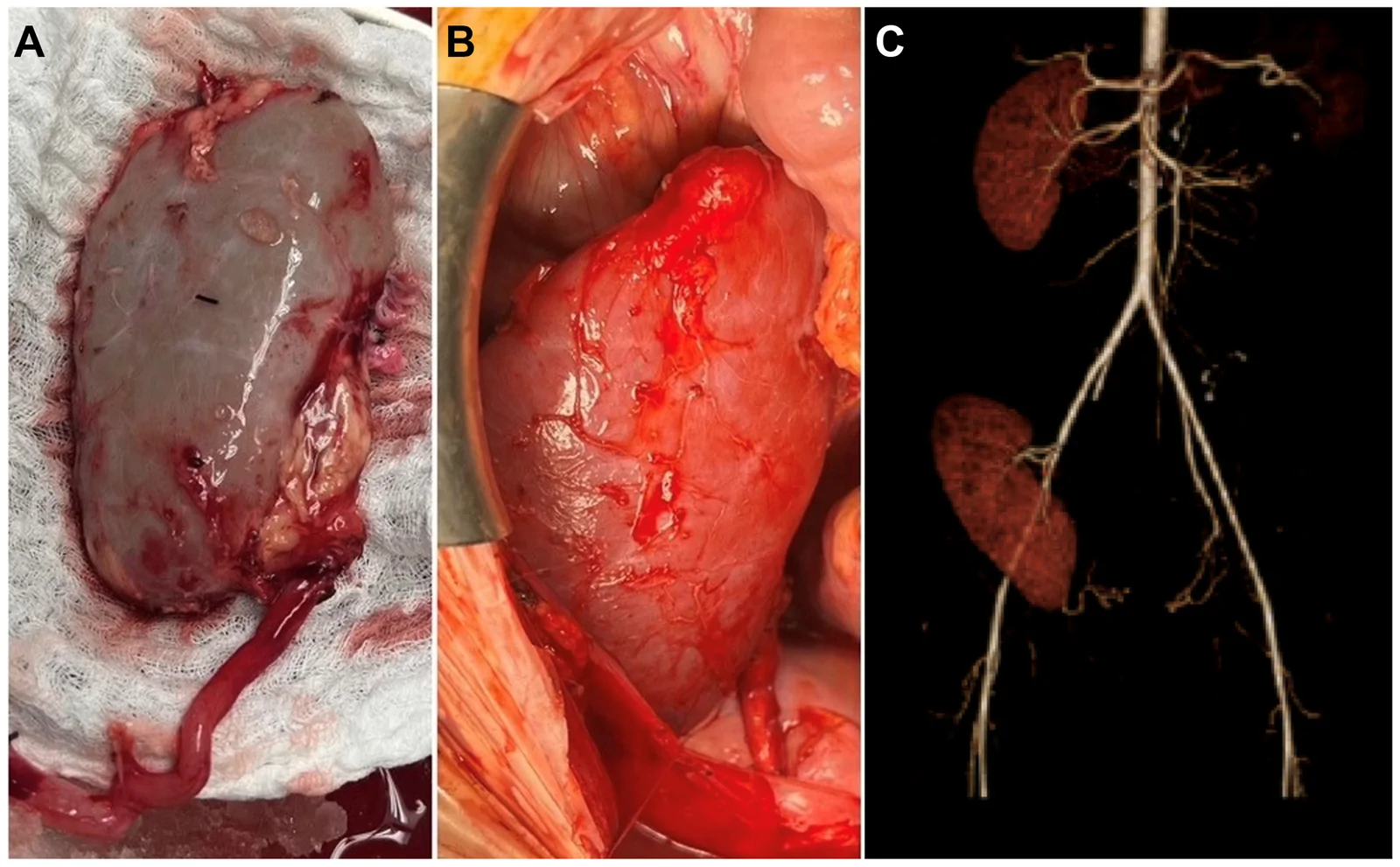
Right transplanted kidney of patient 1. **A,** The resected kidney after back table preparation. **B,** The implanted kidney developed adequate turgor upon reperfusion. **C,** Fifteen months postautotransplantation computed tomography (CT) angiography abdomen and pelvis.

## Case 2

The first patient’s older brother, a 21-year-old man with a history of postural orthostatic tachycardia syndrome, mast cell activation syndrome, hEDS, and MALS presented with chronic abdominal and left-sided flank pain. Computed tomography determined a narrow SMA angle of 9.2 degrees. Venography with IVUS demonstrated compression in the presence of complex circumaortic anatomy, including multiple posterior LRVs and an anterior LRV (Fig 4).

**Fig 4:**
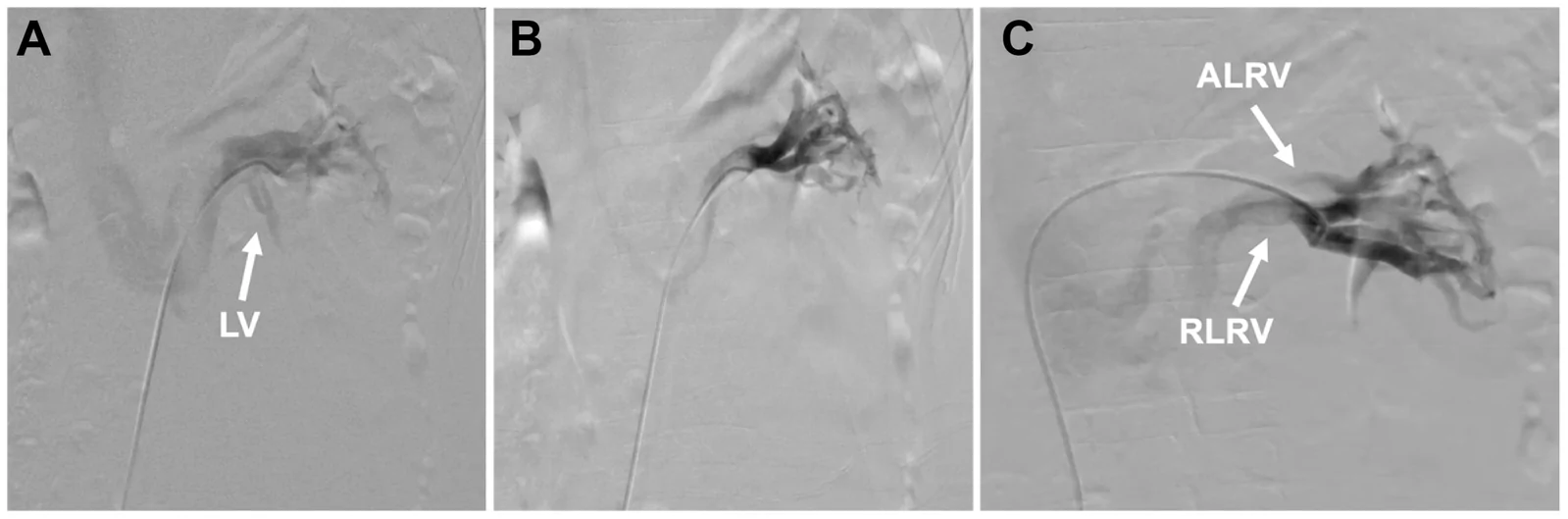
Patient 2 preoperative venogram shows **(A)** large left renal vein (LRV) draining into lumbar vein (LV), **(B** and **C)** multiple retroaortic LRVs (RLRV), and one small anterior LRV (ALRV).

The patient underwent open surgical repair with extensive adhesiolysis and transposition of the dominant posterior LRV. The remaining posterior and anterior renal veins were preserved. This was performed in conjunction with MALS release.

Six months later, the patient developed recurrent left-sided abdominal pain. Repeat venography revealed enlargement of a posterior LRV, a patent anterior LRV, and the development of additional collateral veins (Fig 5). Given persistent symptoms, the patient underwent the multidisciplinary approach to autotransplant as described above. The renal vein, which had multiple branches, was reconstructed into a single venous outflow channel; the two largest branches were anastomosed together to create a common venous conduit, whereas the remaining smaller branches were ligated. After reperfusion, the graft demonstrated immediate urine output. The patient recovered well, postoperatively, and reported improvement of abdominal pain. Duplex ultrasound confirmed a patent LRV (Supplementary Fig, online only). Renal function remained preserved, with a postoperative serum creatinine of 0.80 mg/dL compared with 1.02 mg/dL, preoperatively. At 18 months postoperatively, the transplanted kidney was without hydronephrosis, and the patient reported sustained resolution of nutcracker-associated symptoms and improved quality of life.

**Fig 5:**
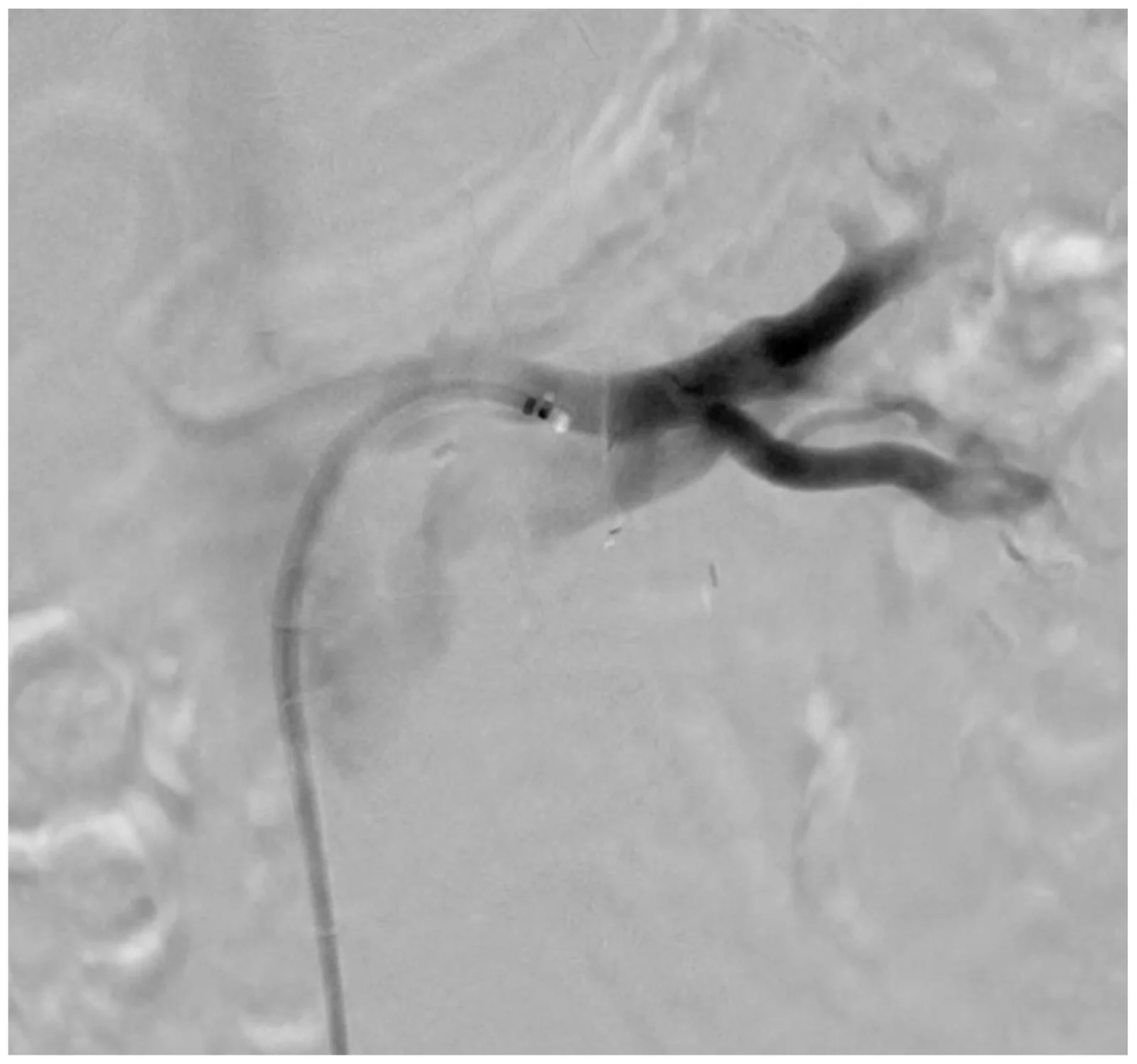
Venography post-left renal vein (LRV) transposition in patient 2 demonstrates extensive collateralization and a narrowed posterior LRV.

## Discussion

In this case series, we described two cases of NCS in siblings with circumaortic LRVs who underwent successful renal autotransplantation after failed LRV transposition. Following autotransplant intervention, they reported resolution of nutcracker-related symptoms with maintained renal function.

Considering the occurrence of this condition in siblings with a shared history of hEDS, there is reason to suspect that there is a genetic component associated with their venous compression. Although genetic testing was not performed with these cases, this remains an important area of further research.

LRV compression in NCS can be treated with several different interventions. In patients with mild-to-moderate disease, conservative measures including weight gain, medication, and close observation are available.^7^ In more severe cases, intervention options include LRV stenting, transposition, and autotransplantation.

Endovascular stenting represents the least invasive treatment option, involving placement of a self-expanding metallic stent within the compressed segment of the LRV.^8^ This approach has demonstrated symptom resolution rates of ∼76%, with a reintervention rate of 11.3%.^7^ Reported complications include stent migration, deployment failure, and stent occlusion.^8^^,^^9^ Endovascular stenting has not been proven effective in the cases of compression with a circumaortic LRV.

LRV transposition has traditionally been regarded as the preferred surgical approach for NCS.^10^^,^^11^ LRV transposition bypasses the site of compression by reimplanting the LRV into the IVC at a lower position and can be performed using open, laparoscopic, or robot-assisted techniques.^12^ This procedure provides an estimated ∼92% rate of symptom resolution, with reintervention required in ∼28% of cases.^7^ Although preferred, its role in posterior NCS with the circumaortic anomaly remains unclear. Existing evidence is limited to case reports and small series, which suggest autotransplantation may be effective as both a primary and salvage intervention.^10^^,^^11^

Autotransplantation is a more invasive surgical option that involves nephrectomy, ex vivo reconstruction, and reimplantation of the kidney. In a meta-analysis of NCS interventions, autotransplantation had the lowest reintervention rate (∼7.2%).^7^ Notably, recent studies evaluating both primary and salvage autotransplantation report higher success rates, ranging from 91% to 93%.^13^^,^^14^ Autotransplantation is considered an effective salvage procedure when other interventions fail to achieve symptom resolution.^14^^,^^15^

In our clinical experience, patients with an aortomesenteric angle <14 degrees often fail LRV transposition due to recurrent compression.^16^ Patients with a posterior LRV may have a higher likelihood of a very narrow SMA angle as presented in these cases. Therefore, we posit their less favorable outcome due to continuous LRV exposure to underlying compressive forces. Removal of the LRV from the aortomesenteric region during autotransplantation avoided recurrent compression and addressed any anatomic constraints brought forth by the circumaortic LRV configuration. However, continued research with future retrospective and prospective studies is needed to best delineate the optimal treatment algorithm for this patient population. The cases presented here contribute to this limited body of literature by further supporting autotransplantation for this complex anatomy.

## Conclusions

Although LRV transposition is considered the gold standard treatment, it failed to relieve symptoms in both cases presented here. In contrast, autotransplantation achieved symptom resolution without significant complications at follow-up. Consistent with emerging literature, these findings suggest that renal autotransplantation may warrant earlier consideration in selected patients with NCS with complex venous anatomy and persistent aortomesenteric configuration. Further studies should directly compare outcomes of primary autotransplantation and LRV transposition in this population.

## Funding

None.

## Disclosures

None.

## Figures and Tables

**Supplementary Fig (online only):**